# Supplementary material for: Chronic loneliness and isolation phenotypes and physical and mental health in older adults
Source: Nat Commun. 2026 Sep 11;17:10730. doi: 10.1038/s41467-026-77683-x (PMC13658241; doi:10.1038/s41467-026-77683-x)
Supplement: Supplementary file 1 [file 41467_2026_77683_MOESM1_ESM.pdf]

Supplementary Materials

Supplementary Figure 1. Measurement of variables across waves

Supplementary Table 1. Proportion of missingness in covariates and outcomes (n=4,654)

Supplementary Table 2. Longitudinal Associations Between Chronicity of Loneliness and Subsequent Physical and Mental Health in Later Life

Supplementary Table 3. Longitudinal Associations Between Chronicity of Isolation and Subsequent Physical and Mental Health in Later Life

Supplementary Table 4. Robustness to unmeasured confounding (E-values) for the longitudinal associations between loneliness, social isolation and physical and mental wellbeing

Supplementary Table 5. Unadjusted Models for Longitudinal Associations Between Chronicity of Loneliness and Subsequent Physical and Mental Health in Later Life

Supplementary Table 6. Unadjusted Models for Longitudinal Associations Between Chronicity of Isolation and Subsequent Physical and Mental Health in Later Life

Supplementary Table 7. Concurrent Associations Between Chronicity of Loneliness and Subsequent Physical and Mental Health in Later Life

Supplementary Table 8. Concurrent Associations Between Chronicity of Isolation and Subsequent Physical and Mental Health in Later Life

Supplementary Figure 1. Measurement of variables across waves

	Pre-wave (wave 6)	Pre-wave (wave 7)	Baseline (wave 8)	Follow-up (wave 9)
**Sociodemographic characteristics**				
Age	X			
Sex	X			
Ethnicity	X			
Education	X			
Employment	X			
Marital status	X			
Total (non-pension) wealth	X			
**Exposures**				
Loneliness		X	X	
Isolation		X	X	
**General health**				
Self-rated health	X		X	X
Number of chronic illnesses	X		X	X
Frequent pain	X		X	X
**Functional capacity**				
ADLs disability	X		X	X
IADLs disability	X		X	X
Mobility disability	X		X	X
Gait speed	X		X	X
**Cognitive function**				
Cognitive functional score		X	X	X
**Mental wellbeing**				
Depressive symptoms	X		X	X
Life satisfaction	X		X	X
Happiness	X		X	X
Quality of life	X		X	X
**Health behaviour**				
Sleep problems	X		X	X
Sedentary behaviour	X		X	X
Smoking	X		X	X
Alcohol consumption	X		X	X
Leisure engagement	X		X	X

Supplementary Table 1. Proportion of missingness in covariates and outcomes (n=4,654)

		**Pre-wave (wave 6)**		**Baseline (wave 8)**		**Follow-up (wave 9)**	
		n	%	n	%	n	%
**Sociodemographic characteristics**							
	Age	7	0.2%				
	Sex	0	0.0%				
	Ethnicity	0	0.0%				
	Education	604	13.0%				
	Employment	199	4.3%				
	Marital status	2	0.0%				
	Total (non-pension) wealth	83	1.8%				
**General health**							
	Self-rated health	16	0.3%	1	0.0%	62	1.3%
	Number of chronic illnesses	1	0.0%	1	0.0%	6	0.1%
	Frequent pain	17	0.4%	4	0.1%	62	1.3%
**Functional capacity**							0.0%
	ADLs disability	0	0.0%	0	0.0%	0	0.0%
	IADLs disability	0	0.0%	0	0.0%	0	0.0%
	Mobility disability	0	0.0%	0	0.0%	0	0.0%
	Gait speed	1200	25.8%	485	10.4%	429	9.2%
**Mental wellbeing**							0.0%
	Depressive symptoms	44	0.9%	29	0.6%	97	2.1%
	Life satisfaction	203	4.4%	38	0.8%	298	6.4%
	Happiness	341	7.3%	119	2.6%	437	9.4%
	Quality of life	392	8.4%	242	5.2%	540	11.6%
**Health behaviour**							0.0%
	Sleep problems	25	0.5%	10	0.2%	72	1.5%
	Sedentary behaviour	0	0.0%	0	0.0%	0	0.0%
	Smoking	0	0.0%	0	0.0%	0	0.0%
	Alcohol consumption	725	15.6%	668	14.4%	275	5.9%
	Leisure engagement	0	0.0%	0	0.0%	0	0.0%
		Pre-wave (wave 7)		Baseline (wave 8)		Follow-up (wave 9)	
**Cognitive function**	Cognitive functional score	184	4.5%	233	5.7%	350	8.6%

Supplementary Table 2. Longitudinal Associations Between Chronicity of Loneliness and Subsequent Physical and Mental Health in Later Life

		**Loneliness**					
**Outcomes**		Transition out of loneliness		Onset of loneliness		Chronic loneliness	
	Coef	Coef [95% CI]	P	Coef [95% CI]	P	Coef [95% CI]	P
**General health**							
Chronic illnesses	OR	1.29 [0.92-1.81]	0.143	1.08 [0.80-1.46]	0.601	**1.59 [1.24-2.04]**	**<0.001**
Chronic pain	OR	1.19 [0.86-1.65]	0.288	**1.39 [1.02-1.89]**	**0.035**	**1.71 [1.33-2.18]**	**<0.001**
Self-rated health	OR	**0.69 [0.49-0.96]**	**0.028**	**0.57 [0.41-0.79]**	**0.001**	**0.45 [0.34-0.60]**	**<0.001**
**Functional capacity**							
IADLs disability	OR	1.34 [0.89-2.03]	0.165	**1.68 [1.17-2.42]**	**0.005**	**1.92 [1.42-2.60]**	**<0.001**
ADLs disability	OR	1.26 [0.81-1.94]	0.307	**1.65 [1.17-2.34]**	**0.004**	**2.09 [1.61-2.73]**	**<0.001**
Mobility limitations	OR	1.14 [0.80-1.63]	0.472	**1.41 [1.02-1.94]**	**0.036**	**1.69 [1.29-2.21]**	**<0.001**
Gait speed	β	-0.11 [-0.24-0.02]	0.094	-0.12 [-0.25-0.02]	0.085	**-0.14 [-0.24- -0.04]**	**0.008**
**Cognitive function**							
Cognitive health	β	-0.08 [-0.20-0.04]	0.191	-0.09 [-0.20-0.01]	0.084	-0.05 [-0.14-0.03]	0.227
**Mental wellbeing**							
Depression	OR	**1.78 [1.24-2.54]**	**0.002**	**1.98 [1.39-2.84]**	**<0.001**	**4.39 [3.36-5.74]**	**<0.001**
Life satisfaction	OR	0.69 [0.48-1.00]	0.051	**0.53 [0.37-0.75]**	**<0.001**	**0.28 [0.21-0.37]**	**<0.001**
Happiness	OR	**0.48 [0.34-0.67]**	**<0.001**	**0.46 [0.33-0.64]**	**<0.001**	**0.25 [0.19-0.32]**	**<0.001**
Quality of life	β	0.07 [-0.07-0.21]	0.331	-0.09 [-0.22-0.04]	0.163	**-0.22 [-0.34- -0.11]**	**<0.001**
**Health behaviour**							
Sleep problems	OR	1.32 [0.96-1.83]	0.089	**1.62 [1.21-2.16]**	**0.001**	**1.91 [1.50-2.42]**	**<0.001**
Sedentary behaviour	OR	1.23 [0.86-1.74]	0.252	**1.63 [1.16-2.28]**	**0.005**	**1.54 [1.16-2.05]**	**0.003**
Smoking	OR	1.23 [0.64-2.37]	0.527	1.08 [0.62-1.88]	0.790	0.87 [0.54-1.38]	0.546
Alcohol consumption	OR	1.07 [0.76-1.51]	0.683	1.11 [0.81-1.50]	0.523	0.78 [0.59-1.03]	0.078
Leisure engagement	β	-0.02 [-0.12-0.08]	0.713	-0.09 [-0.19-0.01]	0.082	-0.03 [-0.11-0.05]	0.465

Note. The reference category was no loneliness. Results were weighted and based on 50 imputed datasets. All models adjusted for age, sex, ethnicity, education, employment, marital status, total net non-pension wealth, and the corresponding pre-baseline outcome measured at wave 6. Models examining cognitive health were adjusted for cognitive scores at wave 7 as potential confounders. Bold text indicates p<0.05. OR: odds ratio from logistic regression model (ordered logistic regression model for alcohol consumption). β: standardized coefficient from linear regression model. All tests were two-sided. No adjustments were made for multiple comparisons.

Supplementary Table 3. Longitudinal Associations Between Chronicity of Isolation and Subsequent Physical and Mental Health in Later Life

		**Social isolation**					
**Outcomes**		Transition out of isolation		Onset of isolation		Chronic isolation	
	Coef	Coef [95% CI]	P	Coef [95% CI]	P	Coef [95% CI]	P
**General health**							
Chronic illnesses	OR	1.05 [0.89-1.25]	0.561	1.19 [0.81-1.74]	0.368	1.16 [0.95-1.43]	0.142
Chronic pain	OR	0.93 [0.78-1.11]	0.434	1.10 [0.77-1.56]	0.607	0.91 [0.74-1.11]	0.340
Self-rated health	OR	1.09 [0.91-1.32]	0.340	0.81 [0.52-1.25]	0.345	1.03 [0.83-1.27]	0.814
**Functional capacity**							
IADLs disability	OR	0.96 [0.75-1.22]	0.730	1.11 [0.69-1.79]	0.666	1.04 [0.79-1.35]	0.790
ADLs disability	OR	1.04 [0.82-1.31]	0.740	1.32 [0.82-2.12]	0.254	1.11 [0.86-1.44]	0.415
Mobility limitations	OR	0.91 [0.76-1.09]	0.309	1.12 [0.73-1.72]	0.591	0.86 [0.70-1.07]	0.178
Gait speed	β	0.01 [-0.05-0.08]	0.721	0.01 [-0.13-0.15]	0.901	-0.04 [-0.12-0.04]	0.350
**Cognitive function**							
Cognitive health	β	**0.07 [0.01-0.13]**	**0.029**	0.03 [-0.08-0.14]	0.640	0.04 [-0.03-0.11]	0.261
**Mental wellbeing**							
Depression	OR	**0.69 [0.54-0.88]**	**0.003**	1.20 [0.80-1.80]	0.380	0.99 [0.77-1.28]	0.955
Life satisfaction	OR	1.19 [0.94-1.51]	0.142	0.76 [0.49-1.18]	0.218	**0.77 [0.60-0.99]**	**0.043**
Happiness	OR	**1.39 [1.12-1.73]**	**0.003**	0.79 [0.53-1.18]	0.243	0.94 [0.74-1.19]	0.603
Quality of life	β	0.01 [-0.06-0.07]	0.880	**-0.18 [-0.33- -0.03]**	**0.021**	**-0.15 [-0.23- -0.06]**	**0.001**
**Health behaviour**							
Sleep problems	OR	0.89 [0.75-1.06]	0.204	0.99 [0.70-1.42]	0.974	0.92 [0.75-1.12]	0.396
Sedentary behaviour	OR	1.01 [0.81-1.26]	0.910	1.22 [0.79-1.88]	0.376	0.87 [0.68-1.10]	0.241
Smoking	OR	1.08 [0.79-1.46]	0.635	1.51 [0.73-3.13]	0.266	1.21 [0.83-1.77]	0.320
Alcohol consumption	OR	1.00 [0.85-1.17]	0.995	0.96 [0.67-1.38]	0.834	0.87 [0.71-1.08]	0.200

Note. The reference category was no isolation. Results were weighted and based on 50 imputed datasets. All models adjusted for age, sex, ethnicity, education, employment, total net non-pension wealth, and the corresponding pre-baseline outcome measured at wave 6. Models examining cognitive health were adjusted for cognitive scores at wave 7 as potential confounders. Bold text indicates $p<0.05$. OR: odds ratio from logistic regression model (ordered logistic regression model for alcohol consumption). β: standardized coefficient from linear regression model. All tests were two-sided. No adjustments were made for multiple comparisons.

Supplementary Table 4. Robustness to unmeasured confounding (E-values) for the longitudinal associations between loneliness, social isolation and physical and mental wellbeing

	**Effect estimates**	**Effect estimates**
**Outcomes**	Chronic loneliness	Chronic social isolation
	E (CI)	E (CI)
**General health**		
Chronic illnesses	2.56 (1.79)	1.59 (1.00)
Chronic pain	2.81 (1.99)	1.43 (1.00)
Self-rated health	3.87 (2.72)	1.21 (1.00)
**Functional capacity**		
IADLs disability	3.25 (2.19)	1.24 (1.00)
ADLs disability	3.60 (2.60)	1.46 (1.00)
Mobility limitations	2.77 (1.90)	1.60 (1.00)
Gait speed	1.53 (1.22)	1.23 (1.00)
**Cognitive function**		
Cognitive health	1.28 (1.00)	1.23 (1.00)
**Mental wellbeing**		
Depression	8.25 (6.18)	1.11 (1.00)
Life satisfaction	6.60 (4.85)	1.92 (1.11)
Happiness	7.46 (5.70)	1.32 (1.00)
Quality of life	1.76 (1.45)	1.54 (1.30)
**Health behaviour**		
Sleep problems	3.23 (2.37)	1.39 (1.00)
Sedentary behaviour	2.45 (1.59)	1.56 (1.00)
Smoking	1.56 (1.00)	1.71 (1.00)
Alcohol consumption	1.88 (1.00)	1.56 (1.00)
Leisure engagement	1.20 (1.00)	NA

Note. E-values for the longitudinal associations between chronic loneliness, social isolation and physical and mental wellbeing. All models adjusted for age, sex, ethnicity, education, employment, marital status (only included in models for loneliness), total net non-pension wealth, and the corresponding pre-baseline outcome measured at wave 6. Models examining cognitive health were adjusted for cognitive scores at wave 7 as potential confounders. Results were weighted and based on 50 imputed datasets. No adjustments were made for multiple comparisons.

Supplementary Table 5. Unadjusted Models for Longitudinal Associations Between Chronicity of Loneliness and Subsequent Physical and Mental Health in Later Life

		**Loneliness**					
**Outcomes**		Transition out of loneliness		Onset of loneliness		Chronic loneliness	
	Coef	Coef [95% CI]	P	Coef [95% CI]	P	Coef [95% CI]	P
**General health**							
Chronic illnesses	OR	**1.58 [1.18-2.12]**	**0.002**	**1.33 [1.01-1.76]**	**0.045**	**1.99 [1.57-2.53]**	**<0.001**
Chronic pain	OR	**1.48 [1.12-1.96]**	**0.006**	**1.71 [1.31-2.24]**	**<0.001**	**2.33 [1.87-2.91]**	**<0.001**
Self-rated health	OR	**0.54 [0.40-0.73]**	**<0.001**	**0.43 [0.32-0.59]**	**<0.001**	**0.33 [0.25-0.42]**	**<0.001**
**Functional capacity**							
IADLs disability	OR	**1.63 [1.17-2.26]**	**0.004**	**2.12 [1.57-2.86]**	**<0.001**	**2.64 [2.08-3.37]**	**<0.001**
ADLs disability	OR	**1.50 [1.06-2.12]**	**0.020**	**1.97 [1.45-2.68]**	**<0.001**	**2.77 [2.17-3.52]**	**<0.001**
Mobility limitations	OR	**1.44 [1.09-1.92]**	**0.011**	**1.68 [1.28-2.21]**	**<0.001**	**2.31 [1.84-2.91]**	**<0.001**
Gait speed	β	**-0.24 [-0.40- -0.08]**	**0.003**	**-0.27 [-0.42- -0.11]**	**0.001**	**-0.33 [-0.46- -0.20]**	**<0.001**
**Cognitive function**							
Cognitive health	β	**-0.21 [-0.38- -0.04]**	**0.015**	**-0.20 [-0.35- -0.05]**	**0.010**	**-0.23 [-0.34- -0.12]**	**<0.001**
**Mental wellbeing**							
Depression	OR	**2.59 [1.88-3.58]**	**<0.001**	**2.78 [2.04-3.80]**	**<0.001**	**7.84 [6.20-9.91]**	**<0.001**
Life satisfaction	OR	**0.43 [0.31-0.61]**	**<0.001**	**0.39 [0.28-0.53]**	**<0.001**	**0.15 [0.12-0.19]**	**<0.001**
Happiness	OR	**0.36 [0.26-0.50]**	**<0.001**	**0.37 [0.27-0.50]**	**<0.001**	**0.16 [0.13-0.20]**	**<0.001**
Quality of life	β	**-0.16 [-0.32- -0.01]**	**0.036**	**-0.32 [-0.46- -0.18]**	**<0.001**	**-0.69 [-0.81- -0.56]**	**<0.001**
**Health behaviour**							
Sleep problems	OR	**1.60 [1.21-2.13]**	**0.001**	**2.04 [1.55-2.67]**	**<0.001**	**2.68 [2.15-3.33]**	**<0.001**
Sedentary behaviour	OR	1.35 [0.98-1.87]	0.066	**1.79 [1.34-2.40]**	**<0.001**	**1.87 [1.48-2.36]**	**<0.001**
Smoking	OR	1.14 [0.86-1.51]	0.372	1.08 [0.83-1.41]	0.566	1.16 [0.94-1.45]	0.171
Alcohol consumption	β	**0.75 [0.58-0.97]**	**0.027**	0.88 [0.68-1.14]	0.342	**0.55 [0.44-0.69]**	**<0.001**
Leisure engagement	OR	**-0.19 [-0.33- -0.05]**	**0.006**	**-0.27 [-0.39- -0.14]**	**<0.001**	**-0.25 [-0.35- -0.15]**	**<0.001**

Note. The reference category was no loneliness. Results were weighted and based on 50 imputed datasets. Bold text indicates p<0.05. OR: odds ratio from logistic regression model (ordered logistic regression model for alcohol consumption). β: standardized coefficient from linear regression model. All tests were two-sided. No adjustments were made for multiple comparisons.

Supplementary Table 6. Unadjusted Models for Longitudinal Associations Between Chronicity of Isolation and Subsequent Physical and Mental Health in Later Life

		**Social isolation**					
**Outcomes**		Transition out of isolation		Onset of isolation		Chronic isolation	
	Coef	Coef [95% CI]	P	Coef [95% CI]	P	Coef [95% CI]	P
**General health**							
Chronic illnesses	OR	1.05 [0.90-1.22]	0.551	1.34 [0.95-1.88]	0.092	**1.48 [1.24-1.76]**	**<0.001**
Chronic pain	OR	1.01 [0.86-1.17]	0.946	1.10 [0.79-1.52]	0.585	**1.20 [1.01-1.42]**	**0.042**
Self-rated health	OR	0.92 [0.79-1.07]	0.281	**0.64 [0.46-0.91]**	**0.013**	**0.64 [0.54-0.76]**	**<0.001**
**Functional capacity**							
IADLs disability	OR	**1.35 [1.10-1.67]**	**0.004**	**1.52 [1.02-2.25]**	**0.039**	**1.93 [1.55-2.40]**	**<0.001**
ADLs disability	OR	**1.28 [1.04-1.58]**	**0.019**	**1.57 [1.04-2.37]**	**0.032**	**1.78 [1.42-2.22]**	**<0.001**
Mobility limitations	OR	1.15 [0.99-1.34]	0.069	1.26 [0.91-1.75]	0.164	**1.44 [1.21-1.71]**	**<0.001**
Gait speed	β	**-0.14 [-0.22- -0.06]**	**<0.001**	-0.12 [-0.29- 0.04]	0.150	**-0.35 [-0.44- -0.25]**	**<0.001**
**Cognitive function**							
Cognitive health	β	**-0.10 [-0.18- -0.02]**	**0.011**	**-0.19 [-0.35- -0.02]**	**0.024**	**-0.25 [-0.34- -0.16]**	**<0.001**
**Mental wellbeing**							
Depression	OR	0.94 [0.76-1.17]	0.579	**1.67 [1.14-2.45]**	**0.009**	**1.88 [1.51-2.33]**	**<0.001**
Life satisfaction	OR	1.03 [0.83-1.28]	0.761	**0.55 [0.37-0.82]**	**0.003**	**0.49 [0.39-0.60]**	**<0.001**
Happiness	OR	**1.23 [1.01-1.50]**	**0.044**	**0.63 [0.43-0.92]**	**0.016**	**0.61 [0.50-0.75]**	**<0.001**
Quality of life	β	-0.07 [-0.15-0.004]	0.062	**-0.41 [-0.59- -0.24]**	**<0.001**	**-0.45 [-0.55- -0.36]**	**<0.001**
**Health behaviour**							
Sleep problems	OR	0.97 [0.83-1.13]	0.677	1.10 [0.79-1.52]	0.578	1.13 [0.95-1.35]	0.165
Sedentary behaviour	OR	**1.52 [1.25-1.83]**	**<0.001**	**1.59 [1.07-2.37]**	**0.023**	**1.80 [1.46-2.21]**	**<0.001**
Smoking	OR	0.90 [0.78-1.06]	0.204	0.99 [0.72-1.37]	0.960	1.02 [0.86-1.21]	0.855
Alcohol consumption	β	**0.86 [0.75-0.99]**	**0.034**	0.74 [0.55-1.00]	0.052	**0.56 [0.47-0.67]**	**<0.001**

Note. The reference category was no isolation. Results were weighted and based on 50 imputed datasets. Bold text indicates p<0.05. OR: odds ratio from logistic regression model (ordered logistic regression model for alcohol consumption). β: standardized coefficient from linear regression model. All tests were two-sided. No adjustments were made for multiple comparisons.

Concurrent Associations

Concurrently, chronic loneliness was associated with higher odds of chronic illness, chronic pain, IADLs/ADLs or mobility disability, depression, sleep problems and sedentary behaviour, with lower likelihoods of better self-rated health, life satisfaction and QoL, happiness, engagement and faster gait speed than those not lonely (Supplementary Table 7). Compared with no loneliness, Individuals with the onset of loneliness had increased odds of chronic pain, IADL disability, mobility limitations, depression, sedentary behaviour and experiencing sleep disturbances, and were concurrently associated with decreased likelihoods of good self-rated health, happiness, life satisfaction and QoL. These mental health-related associations were generally consistent for transitioning out of loneliness. Compared with no isolation, chronic isolation was associated with a lower likelihood of better life satisfaction and QoL (Supplementary Tables 8). Those with onset of isolation had increased odds of living with mobility limitations and depression, with decreased likelihood of experiencing positive psychological wellbeing (life satisfaction, happiness, and QoL). Transitioning out of isolation was associated with reduced odds of depression and increased odds of enhanced happiness.

Supplementary Table 7. Concurrent Associations Between Chronicity of Loneliness and Subsequent Physical and Mental Health in Later Life

		Loneliness					
**Outcomes**		Transition out of loneliness	P	Onset of loneliness	P	Chronic loneliness	P
	Coef	Coef [95% CI]		Coef [95% CI]		Coef [95% CI]	
**General health**							
Chronic illnesses	OR	1.33 [0.96-1.86]	0.091	1.17 [0.85-1.61]	0.344	**1.40 [1.09-1.80]**	**0.008**
Chronic pain	OR	1.14 [0.81-1.60]	0.447	**1.35 [1.00-1.83]**	**0.049**	**1.62 [1.28-2.07]**	**<0.001**
Self-rated health	OR	0.75 [0.52-1.06]	0.104	**0.67 [0.48-0.92]**	**0.013**	**0.62 [0.48-0.81]**	**<0.001**
**Functional capacity**							
IADLs disability	OR	1.31 [0.86-1.99]	0.207	**1.65 [1.07-2.56]**	**0.024**	**1.93 [1.43-2.61]**	**<0.001**
ADLs disability	OR	1.17 [0.75-1.85]	0.486	1.27 [0.83-1.93]	0.266	**2.23 [1.68-2.95]**	**<0.001**
Mobility limitations	OR	**1.46 [1.01-2.13]**	**0.046**	**1.48 [1.08-2.03]**	**0.014**	**1.71 [1.31-2.25]**	**<0.001**
Gait speed	β	-0.08 [-0.20-0.04]	0.203	**-0.17 [-0.31- -0.02]**	**0.022**	**-0.21 [-0.33- -0.10]**	**<0.001**
**Cognitive function**							
Cognitive health	β	-0.05 [-0.16-0.05]	0.342	-0.08 [-0.18-0.03]	0.156	-0.04 [-0.13-0.05]	0.369
**Mental wellbeing**							
Depression	OR	**1.54 [1.02-2.30]**	**0.038**	**3.47 [2.48-4.85]**	**<0.001**	**5.23 [3.96-6.89]**	**<0.001**
Life satisfaction	OR	**0.61 [0.42-0.89]**	**0.010**	**0.25 [0.18-0.34]**	**<0.001**	**0.19 [0.15-0.25]**	**<0.001**
Happiness	OR	**0.53 [0.38-0.73]**	**<0.001**	**0.28 [0.21-0.39]**	**<0.001**	**0.22 [0.17-0.28]**	**<0.001**
Quality of life	β	-0.02 [-0.13-0.09]	0.740	**-0.27 [-0.39- -0.16]**	**<0.001**	**-0.38 [-0.49- -0.26]**	**<0.001**
**Health behaviour**							
Sleep problems	OR	1.29 [0.93-1.78]	0.133	**1.73 [1.28-2.35]**	**<0.001**	**2.03 [1.58-2.60]**	**<0.001**
Sedentary behaviour	OR	1.12 [0.78-1.62]	0.527	**1.80 [1.26-2.57]**	**0.001**	**1.51 [1.13-2.01]**	**0.006**
Smoking	OR	1.45 [0.74-2.85]	0.277	1.35 [0.79-2.32]	0.274	1.02 [0.61-1.70]	0.951
Alcohol consumption	OR	0.95 [0.68-1.32]	0.741	0.94 [0.70-1.27]	0.701	0.81 [0.63-1.06]	0.121
Leisure engagement	β	-0.01 [-0.12-0.09]	0.809	-0.04 [-0.13-0.05]	0.341	**-0.12 [-0.19- -0.04]**	**0.002**

Note. The reference category was no loneliness. Results were weighted and based on 50 imputed datasets. All models adjusted for age, sex, ethnicity, education, employment, marital status, total net non-pension wealth, and the corresponding pre-baseline outcome measured at wave 6. Models examining cognitive health were adjusted for cognitive scores at wave 7 as potential confounders. Bold text indicates $p<0.05$. OR: odds ratio from logistic regression model (ordered logistic regression model for alcohol consumption). β: standardized coefficient from linear regression model. All tests were two-sided. No adjustments were made for multiple comparisons.

Supplementary Table 8. Concurrent Associations Between Chronicity of Isolation and Subsequent Physical and Mental Health in Later Life

		Social isolation					
**Outcomes**		Transition out of isolation		Onset of isolation		Chronic isolation	
	Coef	Coef [95% CI]	P	Coef [95% CI]	P	Coef [95% CI]	P
**General health**							
Chronic illnesses	OR	1.08 [0.90-1.30]	0.385	0.85 [0.58-1.23]	0.380	1.17 [0.95-1.44]	0.130
Chronic pain	OR	1.02 [0.85-1.22]	0.817	1.12 [0.76-1.64]	0.572	1.06 [0.86-1.29]	0.604
Self-rated health	OR	1.17 [0.97-1.40]	0.094	0.71 [0.46-1.08]	0.108	0.98 [0.79-1.21]	0.849
**Functional capacity**							
IADLs disability	OR	1.12 [0.87-1.45]	0.375	1.62 [0.95-2.77]	0.074	1.23 [0.93-1.64]	0.143
ADLs disability	OR	0.93 [0.73-1.20]	0.594	1.26 [0.73-2.17]	0.407	1.21 [0.92-1.59]	0.180
Mobility limitations	OR	0.87 [0.73-1.04]	0.134	**1.55 [1.03-2.31]**	**0.034**	0.88 [0.71-1.10]	0.266
Gait speed	β	0.003 [-0.06-0.07]	0.917	-0.07 [-0.22-0.09]	0.414	-0.07 [-0.15-0.02]	0.136
**Cognitive function**							
Cognitive health	β	0.02 [-0.04-0.08]	0.474	0.02 [-0.10-0.14]	0.749	-0.004 [-0.07-0.06]	0.909
**Mental wellbeing**							
Depression	OR	**0.68 [0.53-0.88]**	**0.003**	**1.88 [1.28-2.75]**	**0.001**	0.96 [0.74-1.25]	0.788
Life satisfaction	OR	1.19 [0.93-1.52]	0.175	**0.38 [0.25-0.58]**	**<0.001**	**0.57 [0.45-0.73]**	**<0.001**
Happiness	OR	**1.42 [1.15-1.75]**	**0.001**	**0.60 [0.41-0.87]**	**0.007**	0.88 [0.70-1.10]	0.259
Quality of life	β	0.03 [-0.04-0.09]	0.392	**-0.22 [-0.37- -0.07]**	**0.003**	**-0.19 [-0.27- -0.11]**	**<0.001**
**Health behaviour**							
Sleep problems	OR	0.92 [0.77-1.11]	0.393	1.21 [0.83-1.77]	0.310	0.96 [0.78-1.18]	0.694
Sedentary behaviour	OR	0.92 [0.74-1.16]	0.490	1.06 [0.67-1.66]	0.807	0.92 [0.71-1.18]	0.500
Smoking	OR	1.00 [0.72-1.38]	0.979	1.17 [0.48-2.80]	0.733	1.08 [0.71-1.63]	0.716
Alcohol consumption	OR	0.99 [0.85-1.16]	0.906	1.08 [0.76-1.54]	0.661	0.91 [0.75-1.11]	0.353

Note. The reference category was no isolation. Results were weighted and based on 50 imputed datasets. All models adjusted for age, sex, ethnicity, education, employment, total net non-pension wealth, and the corresponding pre-baseline outcome measured at wave 6. Models examining cognitive health were adjusted for cognitive scores at wave 7 as potential confounders. Bold text indicates $p<0.05$. OR: odds ratio from logistic regression model (ordered logistic regression model for alcohol consumption). β: standardized coefficient from linear regression model. All tests were two-sided. No adjustments were made for multiple comparisons.
