# Supplementary material for: Chronic loneliness and isolation phenotypes and physical and mental health in older adults
Source: Nat Commun. 2026 Sep 11;17:10730. doi: 10.1038/s41467-026-77683-x (PMC13658241; doi:10.1038/s41467-026-77683-x)
Supplement: Supplementary file 2 [file 41467_2026_77683_MOESM2_ESM.pdf]

nature portfolio

Corresponding author(s): Qian Gao

Last updated by author(s): Aug 8, 2026

Reporting Summary

Nature Portfolio wishes to improve the reproducibility of the work that we publish. This form provides structure for consistency and transparency in reporting. For further information on Nature Portfolio policies, see our Editorial Policies and the Editorial Policy Checklist.

Statistics

For all statistical analyses, confirm that the following items are present in the figure legend, table legend, main text, or Methods section.

n/a	Confirmed	
☐	☒	The exact sample size (*n*) for each experimental group/condition, given as a discrete number and unit of measurement
☐	☒	A statement on whether measurements were taken from distinct samples or whether the same sample was measured repeatedly
☐	☒	The statistical test(s) used AND whether they are one- or two-sided<br>*Only common tests should be described solely by name; describe more complex techniques in the Methods section.*
☐	☒	A description of all covariates tested
☐	☒	A description of any assumptions or corrections, such as tests of normality and adjustment for multiple comparisons
☐	☒	A full description of the statistical parameters including central tendency (e.g. means) or other basic estimates (e.g. regression coefficient) AND variation (e.g. standard deviation) or associated estimates of uncertainty (e.g. confidence intervals)
☐	☒	For null hypothesis testing, the test statistic (e.g. *F*, *t*, *r*) with confidence intervals, effect sizes, degrees of freedom and *P* value noted<br>*Give P values as exact values whenever suitable.*
☒	☐	For Bayesian analysis, information on the choice of priors and Markov chain Monte Carlo settings
☒	☐	For hierarchical and complex designs, identification of the appropriate level for tests and full reporting of outcomes
☐	☒	Estimates of effect sizes (e.g. Cohen's *d*, Pearson's *r*), indicating how they were calculated

Our web collection on statistics for biologists contains articles on many of the points above.

Software and code

Policy information about availability of computer code

Data collection	No software was used for data collection
Data analysis	No custom code was developed. Data analyses were performed using R software version 4.3.2 and Stata 17 (StatCorp LP, Texas, USA). Code is available from https://doi.org/10.5281/zenodo.21843961.

For manuscripts utilizing custom algorithms or software that are central to the research but not yet described in published literature, software must be made available to editors and reviewers. We strongly encourage code deposition in a community repository (e.g. GitHub). See the Nature Portfolio guidelines for submitting code & software for further information.

Data

Policy information about availability of data

All manuscripts must include a data availability statement. This statement should provide the following information, where applicable:

- Accession codes, unique identifiers, or web links for publicly available datasets
- A description of any restrictions on data availability
- For clinical datasets or third party data, please ensure that the statement adheres to our policy

ELSA data are publicly available through registration with the UK data service (https://beta.ukdataservice.ac.uk/datacatalogue/series/series?id=200011).

Research involving human participants, their data, or biological material

Policy information about studies with human participants or human data. See also policy information about sex, gender (identity/presentation), and sexual orientation and race, ethnicity and racism.

Reporting on sex and gender	Only sex is collected within ELSA. We reported the distribution of sex in Table 1. The fully adjusted models have adjusted for sex and other confounders (Supplementary Table 2&5)
Reporting on race, ethnicity, or other socially relevant groupings	The study reported the distribution of participants' age (a continuous variable), sex (male versus female), ethnicity (White versus non-White), educational level (no or basic qualifications, GCSE or O-level or qualification at age 16, A-levels or higher education or qualification at age 18, degree or further higher qualification), marital status (married versus not), total net non-pension wealth (quantiles), and employment status (working versus not working)
Population characteristics	The study included 4,090 participants with a mean age of 66.7 years (SE=0.13, age range= 54-89 years) (Table 1). The sample comprised 2,221 women (54.3%) and was predominantly White (3,971 participants, 97.1%), with 119 non-White participants (2.9%). Overall, 2,896 (70.8%) participants were married, and 1,117 participants (27.3%) had only basic education, with 70.1% of the sample being unemployed or retired. Full population characteristics are provided in Table 1 of the manuscript.
Recruitment	ELSA is a longitudinal study including a sample representative of people aged 50 and over living in private households in England. ELSA began in 2002, with follow-up waves conducted every two years. This study is a secondary data analysis of ELSA, and the detailed information about data collection has been described elsewhere (see data availability source in the manuscript).
Ethics oversight	ELSA Wave 9 received ethical approval from the South Central—Berkshire Research Ethics Committee on 10 May 2018 (17/SC/0588). ELSA Wave 8 received ethical approval from the South Central—Berkshire Research Ethics Committee on 23 September 2015 (15/SC/0526). ELSA Wave 7 received ethical approval from the National Research Ethics Service (NRES) Committee South Central—Berkshire on 28 November 2013 (13/SC/0532). ELSA Wave 6 received ethical approval from the NRES Committee South Central—Berkshire on 28 November 2012 (11/SC/0374). All participants provided informed written consent.

Note that full information on the approval of the study protocol must also be provided in the manuscript.

Field-specific reporting

Please select the one below that is the best fit for your research. If you are not sure, read the appropriate sections before making your selection.

☐ Life sciences ☒ Behavioural & social sciences ☐ Ecological, evolutionary & environmental sciences

For a reference copy of the document with all sections, see nature.com/documents/nr-reporting-summary-flat.pdf

Behavioural & social sciences study design

All studies must disclose on these points even when the disclosure is negative.

Study description	Quantitative secondary data analysis of cohort study to assess the physical, mental, behavioural, and cognitive health consequences of chronic loneliness and isolation phenotypes and the transitions between loneliness/social isolation states (i.e. onset and remission) over two years.
Research sample	Representative sample of adults over the age of 50 living in England. Details of the study sample selection process are shown in Figure 4.
Sampling strategy	Random. Our data were derived from the English Longitudinal Study of Ageing (ELSA). ELSA was designed to recruit a representative sample of adults aged 50 years and older living in private households in England. The original sample was drawn from participants from the Health Survey in England in 1998, 1999 and 2001. Further information on the sampling strategy can be found in the data resource.
Data collection	This study is a secondary data analysis of the ELSA, and no data collection was involved in this study. Data collection procedures can be found in the data resource.
Timing	This longitudinal study used panel data from the English Longitudinal Study of Ageing (ELSA). ELSA began in 2002, with follow-up waves conducted every two years. We used ELSA data from the most recent six-year period, including wave 6 (2012-2013), wave 7 (2014–2015), wave 8 (2016–2017) and wave 9 (2018–2019).
Data exclusions	The study excluded participants who did not respond to measures of primary exposure (loneliness and social isolation at waves 7 and 8). Of these participants, we further limited the sample to those with longitudinal weights provided by ELSA, forming a final sample of 4,090 older adults. The missingness was largely due to loss to follow-up and non-response. The exclusion criteria were pre-established.
Non-participation	Of the 7,289 core participants in ELSA at wave 9, we limited our analytical sample to those who participated in all included waves (6-9; n=5,651; 78.2%). In accordance with the primary exposure measures, we then restricted the sample to participants who had

complete measures of loneliness and social isolation at waves 7 and 8 (n=4,654). Of these participants, we further limited the sample to those with longitudinal weights provided by ELSA, forming a final sample of 4,090 older adults. The details of sample selection are displayed in Figure 4.

Randomization	This is a cohort study, so allocation was not random. All models adjusted for the same set of sociodemographic confounders, including age, sex, ethnicity, education, employment, marital status (only included in models for loneliness), and total net non-pension wealth, and also accounted for each outcome from pre-baseline.

Reporting for specific materials, systems and methods

We require information from authors about some types of materials, experimental systems and methods used in many studies. Here, indicate whether each material, system or method listed is relevant to your study. If you are not sure if a list item applies to your research, read the appropriate section before selecting a response.

Materials & experimental systems

n/a	Involved in the study
☒	☐ Antibodies
☒	☐ Eukaryotic cell lines
☒	☐ Palaeontology and archaeology
☒	☐ Animals and other organisms
☒	☐ Clinical data
☒	☐ Dual use research of concern
☒	☐ Plants

Methods

n/a	Involved in the study
☒	☐ ChIP-seq
☒	☐ Flow cytometry
☒	☐ MRI-based neuroimaging

Plants

Seed stocks	N/A
Novel plant genotypes	N/A
Authentication	N/A
