# Supplementary material for: Chronic loneliness and isolation phenotypes and physical and mental health in older adults
Source: Nat Commun. 2026 Sep 11;17:10730. doi: 10.1038/s41467-026-77683-x (PMC13658241; doi:10.1038/s41467-026-77683-x)
Supplement: Supplementary file 3 [file 41467_2026_77683_MOESM3_ESM.pdf]

nature portfolio

Peer Review File

Chronic loneliness and isolation phenotypes and physical and mental health in older adults

Corresponding Author: Dr Qian Gao

This file contains all reviewer reports in order by version, followed by all author rebuttals in order by version.

Version 0:

Reviewer comments:

Reviewer #1

(Remarks to the Author)
Interesting paper.

My comments:
Title is appropriate.
Abstract: Why not reporting SD (plus range) for age?
Abstract: Please clarify how the key variables (chronic loneliness and isolation in particular) were quantified
Abstract: Please clarify the analytical approach used.
Abstract: Please tone down the last sentence. This part is severely overstated
Introduction: what is an „extended period of time“? What is chronic loneliness? What is a „long-term exposure to loneliness“?
Introduction: The added value should be clarified in more detail. For example, what is the added value compared to Ref. 5? (which has a very similar title and a very similar content)
The sentence „pathways underlying the associations… have yet to be explored“ sounds like a mediation approach.
Introduction: The last sentence is severely overstated. Please tone down.
Results: First sentence: „SD=0.13“? You probably refer to SE.
Results: Please distinguish between retirement and unemployment.
Results: Depression or probable depression?
Results: Please avoid the term „effects“ (e.g., „Onset of loneliness showed similar effects for IADL disability“)
Results: Why is life satisfaction „mental wellbeing“ (same question: happiness -> mental wellbeing)?
On which theoretical model is this classification based?
E-value anlayses: well done!
Please correct typos (e.g., „cognative ability“)
Strengths and limitations: Mainly appropriate.
Discussion: Please focus on the magnitude of the associations.
Conclusion: Please tone down the wording. Moreover, please tone down the practical implications. Reverse causality cannot be ruled out.
Methods: Please add response rates for the waves used (and attrition rate)
Methods: Why just a binary indicator of chronic conditions? (most older individuals have at least two chronic conditions)?
Why only these few chronic diseases?
Why just a single-item to quantify pain?
Please add psychometric properties for the cognitive function tool.
Depression: Please change to probable depression. This is just a screening tool.
Life satisfaction: Why just a single-item tool? Why dichotomize it?
Health behaviors: Why outcomes? Could these not rather be potential mediators?
Covariates: Why these specific covariates? This remains unclear. Why not adjusting for, e.g., personality?
Statistical analysis: „All models adjusted for… and also account for reach outcome from pre-baseline“.
The use of lagged dependent variables (LDVs) as covariates in regression models can lead to bias (Nickell Bias). Please justify this choice.
25.8% (missingness, gait speed). This can be a serious issue. PLease describe the MI approach in much more detail.
Why do so many people not disclose their education or alcohol consumption?
Why isn't the syntax provided by default?

Supplementary Tables are helpful. Thank you. Please also report the covariates there.

Reviewer #2

(Remarks to the Author)
Thank you for the opportunity to review the paper entitled ‘Chronic loneliness and isolation phenotypes and physical and mental health in older adults’. The study aims to assess the association between chronic loneliness and chronic social isolation and 17 health outcomes. It adds to the growing body of research which focuses on chronic loneliness, in particular. The exposures, chronic loneliness and chronic social isolation, were defined as being lonely or isolated at two consecutive data time points. While there is not an agreed upon measure of chronic or transient loneliness or isolation, prior research generally refers to chronic loneliness or isolation as being persistent, lasting for several years or more or at consecutive time points.

Chronic loneliness was associated with increased odds of poorer health, mental wellbeing, functional capacity and health behaviours. Chronic isolation was associated with lower quality of life, and transient or remitting social isolation was associated with better mental wellbeing and cognitive function. Supplementary analysis indicates that the main results hold when both loneliness and isolation are included in the same model.

I just have a few additional comments:
• Most of the focus of the introduction is chronic loneliness. More could be developed about social isolation and the potential implications of chronic social isolation.
• I suggest the authors consider some of the recent papers on chronic loneliness and chronic social isolation (see for example, the review by Hajek et al. (2025). Chronic loneliness and chronic social isolation among older adults. A systematic review, meta-analysis and meta-regression, Aging & Mental Health, 29:2, 185-200, DOI: 10.1080/13607863.2024.2385448; Lim, M.H., Manera, K.E., Owen, K.B. et al. (2023) The prevalence of chronic and episodic loneliness and social isolation from a longitudinal survey. Scientific Reports, 13, 12453. https://doi.org/10.1038/s41598-023-39289-x)
• The findings for social isolation and health behaviours are not commented on.

Reviewer #3

(Remarks to the Author)
I am afraid that i found the paper somewhat hard to understand. It would help if the Methods section was in the correct place - it appears to be placed after the discussion. However, I think what is missing is a clear definition of the exposures - chronic loneliness and chronic social isolation - transient loneliness etc. You need to say how these were determined and analysed. It would then also help if you provided very clear research questions and/or hypotheses.

Reviewer #4

(Remarks to the Author)
This manuscript provides a comprehensive analysis using ELSA data to study the associations between chronic loneliness and isolation and physical and mental health in older adults. Overall, the manuscript effectively reflected on prior literature and addresses an important topic substantively. Below, I provide a mix of major and minor critiques for your consideration.
Major Comments:
1. In the Background (p.2), the authors suggest that “older people are particularly vulnerable to loneliness”. However, the youngest age groups are more likely to report loneliness than other age groups (e.g., Office for National Statistics, 2025). Although, older people are more likely to have experiences that lead to loneliness, authors need to make more accurate claims.
2. Authors also suggested that “health effects of loneliness are more prominent among older adults” (p.2). Can the authors provide a reference to support this claim?
3. The mechanisms behind the associations between loneliness and physical health are discussed (p.2). As the manuscript also focuses on mental health, can the authors discuss the ways how chronic loneliness may affect mental health (i.e. depression and cognitive function changes)?
4. It would be helpful to provide a figure that makes it clear what variables were measured at each wave.
5. The cut-off of ≥3 for CES-D score was used in the study to indicate depression. Many studies have also used cut-off ≥4 to indicate potential depression (e.g.,Beach et al., 2003, PLOS Medicine). Have authors conducted the analysis with cut-off 4 to compare if there were any differences in the results?
Minor comments:
1. When discussion the limitations, the authors could also mention healthy survival bias, which is relevant for the study.
2. Can the authors provide the references for the use of ADLs, IADLs, functional difficulties, and sedentary behaviour measures (p.14)?
3. In the sensitivity analysis section (p. 7), can the authors provide a reference to support the claim that the results were moderately robust to unmeasured confounding, according to the calculated E-values?
4. In the Supplementary Figure 1, the number of participants not included in all waves seems to be missing.

Version 1:

Reviewer comments:

Reviewer #1

(Remarks to the Author)
For the most part, the issues were addressed effectively.

A few issues:
Abstract: The last sentence is still phrased in a very causal manner. Please revise it further. Endogeneity cannot be ruled out. The conclusions are also too strong.
Conclusion (text): Please tone down (see my comment above).
Lagged dependent variable: Please consider omitted variable bias. The LDV is very likely correlated with unobserved heterogeneity (e.g., personality, genetic factors). The model then incorrectly attributes
an influence to the lagged value that actually stems from these stable factors. Please extend the limitations section accordingly (or modify the model)
Why isn't the Stata code (I don't mean the DTA file) made available (but only upon request)? Unfortunately, this question isn't clearly answered. In the spirit of open science, this would be highly desirable.

Reviewer #2

(Remarks to the Author)
The authors have addressed my previous comments and the paper has improved.

Reviewer #3

(Remarks to the Author)
I have several concerns about this paper:

You ought to demonstrate how this study clearly differs from other studies of loneliness and health using the ELSA data. I have a strong sense of deja vu; I feel I have seen many of these findings before - thus, you need to state what is different or innovative.

There is no methods section to state how you arrived at your findings.
The research questions are not stated
There are no hypotheses to guide this work
Might be useful to offer a power calculation

minor but important point - never use the term "non-white" - first this is not an ethnic category and second, it always feels pejorative.

Reviewer #4

(Remarks to the Author)
Thank you for addressing the comments. I am satisfied with the responses and revisions made to the manuscript.

Version 2:

Reviewer comments:

Reviewer #1

(Remarks to the Author)
Thank you for further improving the paper.

However, one point definitely needs to be worded more carefully.
More precisely, please avoid the term "dose-response relationship" (which sounds very causal) in the abstract and the conclusion (main text).
For example, you could rephrase it to: "is associated with"

Reviewer #3

(Remarks to the Author)
I am satisfied that the authors have addressed the main comments.

Open Access This Peer Review File is licensed under a Creative Commons Attribution 4.0 International License, which permits use, sharing, adaptation, distribution and reproduction in any medium or format, as long as you give appropriate credit to the original author(s) and the source, provide a link to the Creative Commons license, and indicate if changes were made.
In cases where reviewers are anonymous, credit should be given to 'Anonymous Referee' and the source.
The images or other third party material in this Peer Review File are included in the article's Creative Commons license, unless indicated otherwise in a credit line to the material. If material is not included in the article's Creative Commons license and your intended use is not permitted by statutory regulation or exceeds the permitted use, you will need to obtain permission directly from the copyright holder.
To view a copy of this license, visit https://creativecommons.org/licenses/by/4.0/

Dear Editor,

Thank you for the opportunity to revise our manuscript.

We are grateful to the editor and reviewers for their encouraging and insightful comments, all of which we have carefully attended to and responded to below. We therefore hope this paper is now suitable for acceptance.

The authors

REVIEWER'S COMMENTS

Reviewer #1

Comment 1: Interesting paper. Title is appropriate.
Abstract: Why not reporting SD (plus range) for age?
Response 1: Thank you. All analyses were conducted on imputed data and weighted using longitudinal weights to account for sample attrition and complex sampling design. So we followed the standard practice by reporting weighted means and associated standard errors (SE), since SDs do not adequately account for the uncertainty introduced by weighting and imputation. We have now added the age range (54-89 years) to both the abstract and results sections (Page 3).

Comment 2: Abstract: Please clarify how the key variables (chronic loneliness and isolation in particular) were quantified
Response 2: Thank you for noting that this was not clear. We have now clarified the measure in abstract: "Chronic social deficits were defined as sustained exposure to loneliness or isolation across two consecutive waves of the cohort (spanning two years)."

Comment 3: Abstract: Please clarify the analytical approach used.
Response 3: We have now clarified the analytical approach used in the Abstract: "Using regression models, we assessed how the chronicity of social deficits was associated with 17 health outcomes across physical, mental, cognitive and behavioural health domains."

Comment 4: Abstract: Please tone down the last sentence. This part is severely overstated
Response 4: We have now revised it as: "Findings suggest a potential dose-response relationship between chronicity of loneliness and adverse health outcomes, indicating that addressing chronic social deficits may contribute to supporting healthy ageing."

Comment 5: Introduction: what is an „extended period of time"? What is chronic loneliness? What is a „long-term exposure to loneliness"?
Response 5: Previous research has examined chronic loneliness and social isolation, yet no consensus exists on a precise threshold for this phenotype. Studies commonly measure it as loneliness/isolation reported consistently across repeated assessments over years. We have now cited recent publications on chronic loneliness/isolation. In our study, we initially used the term "long-term exposure to loneliness" as a proxy for chronic loneliness, but we have now updated the terminology to "chronic loneliness" for clarity.

We have now revised this part of the Introduction, in accordance with the most widely adopted definition of chronic loneliness/isolation: "Chronic loneliness and isolation phenotypes present as a persistent, lasting emotional state of feeling lonely or disconnected over an extended period of time [5,8,18–20]. In contrast, transient loneliness or isolation represents a subacute phenotype, with different health consequences compared to the persistent trait [21]. Although there is no consensus threshold for the chronic phenotype, studies commonly define it as loneliness or isolation reported consistently across repeated measurements over years or at consecutive time points [5,8,20]." (Page 2).

Comment 6: Introduction: The added value should be clarified in more detail. For example, what is the added value compared to Ref. 5? (which has a very similar title and a very similar content)
The sentence „pathways underlying the associations… have yet to be explored“ sounds like a mediation approach.
Response 6: Ref. 5 investigated the role of chronic loneliness and isolation in predicting mortality as well as the onset of difficulties with activities of daily living (ADL) as a competing outcome in modelling. Our study builds on this existing evidence by exploring the various physical, mental, and behavioural pathways that might underlie this association between chronic loneliness and mortality. We use an outcome-wide approach to look at a diverse range of physical, mental, and behavioural factors, taking the first step in identifying which are associated with chronic loneliness. This could lead to future research exploring whether specific factors are mediators of the association between chronic loneliness and mortality. We are testing a range of factors that could represent potential mechanisms. The value comes both from 1) identifying the outcomes of chronic phenotypes so that we better understand how chronic loneliness/isolation might be influencing health, and 2) identifying potentially modifiable mediators of the relationship between chronic loneliness/isolation and mortality that could be further tested in future research and ultimately intervened on.

We have now revised the introduction as: "Although chronic loneliness and social isolation have been identified as independent risk factors for premature mortality in older age [8,15,23], the physical, mental and behavioural pathways underlying the associations between chronic social deficits and premature mortality have yet to be fully explored [5]. Further research should build on this evidence by testing a range of factors that could represent potential mechanisms. This could help explore the health outcomes of chronic phenotypes and identify potential modifiable mediators of their association with mortality that could be further examined and ultimately intervened on.

Thus, this study used an outcome-wide approach to assess the physical, mental, behavioural, and cognitive health consequences of chronic loneliness and isolation phenotypes and the transitions between loneliness/social isolation states (i.e. onset and remission) over two years. We hypothesised that chronic and incident loneliness/isolation would be associated with worse health outcomes, whereas remission would be linked to improvements. This will inform future research to potentially reveal mechanisms through which chronic social deficits could lead to increased mortality." (Page 3).

Comment 7: Introduction: The last sentence is severely overstated. Please tone down.
Response 7: We have now revised it as: "This will inform future research to potentially reveal mechanisms through which chronic social deficits could lead to increased mortality."

Comment 8: Results: First sentence: „SD=0.13“? You probably refer to SE.
Response 8: Thank you for pointing out this error. We have now corrected it.

Comment 9: Results: Please distinguish between retirement and unemployment.
Response 9: We used "retirement and unemployment" to describe 'not working'. This confounder is a binary measure in the survey, captured by the original question "Whether respondent was in paid employment last month [yes/no]". We have now corrected "retirement and unemployment" to "not working" for our employment-status measure (categorised as working vs not working) to avoid potential misinterpretation. We retained categorisation to ensure consistency with previous publications on chronic loneliness and social isolation using ELSA data (e.g. https://www.nature.com/articles/s44220-025-00436-0). Additionally, we estimated E-values to assess robustness to residual confounding, which indicated acceptable robustness.

Comment 10: Results: Depression or probable depression?
Response 10: Thank you for noting that this was unclear. The eight-item Center for Epidemiologic Studies Depression Scale (CES-D) is a well-established, validated measure that is widely used in survey data and population-based studies of late-life depression (doi:10.1097/JGP.0b013e3181dba6d2,

https://doi.org/10.1016/S2215-0366(16)00097-3, https://doi.org/10.1038/s41591-023-02506-1, etc.). Whilst it cannot measure clinical diagnoses, it is the best instrument for measuring depression available in ELSA. We have clarified that "depression" is operationalised in our study as "probable depression (above-threshold depressive symptom scores)"(Page 16) and have acknowledged the potential limitation in the paper: "Depression was measured using the validated CES-D scale, but we did not examine the impact of psychiatric diagnoses on our findings." (Page 13). We have now revised depression to probable depression throughout the Results.

Comment 11: Results: Please avoid the term „effects" (e.g., „Onset of loneliness showed similar effects for IADL disability")
Response 11: We have removed the word effects throughout the results section. For example, we have now revised this sentence: "Onset of loneliness was similarly associated with IADL disability (OR=1.68, 95%CI=1.17-2.42), ADL disability (OR=1.65, 95%CI=1.17-2.34), and mobility limitations (OR=1.41, 95%CI=1.02-1.94)."; "Onset of loneliness was associated with significant but less pronounced higher odds of probable depression (OR=1.98, 95%CI=1.39-2.84), and lower odds of life satisfaction (OR=0.53, 95%CI=0.37-0.75), and happiness (OR=0.46, 95%CI=0.33-0.64)" (Page 7).

Comment 12: Results: Why is life satisfaction „mental wellbeing" (same question: happiness -> mental wellbeing)? On which theoretical model is this classification based?
Response 12: In the manuscript, we follow the common conceptualisation of subjective wellbeing as a key component of mental wellbeing. Mental wellbeing is a multidimensional construct that includes both cognitive and affective components. Life satisfaction reflects the evaluative dimension of wellbeing (how individuals appraise their life as a whole), whereas happiness reflects the affective dimension, relating to positive emotions and feelings. Both constructs are widely treated as core indicators of established wellbeing frameworks (e.g. subjective wellbeing models [ref 65]). However, we acknowledge that these are indicators of subjective mental wellbeing rather than comprehensive assessments. In this study, we conveniently grouped both measures in mental wellbeing domain primarily to help organise and summarise the wide range of outcomes.

We have now clarified this in the Methods: "In accordance with the subjective wellbeing model [65], we grouped life satisfaction and happiness under the broader domain of mental wellbeing." (Page 16)

Comment 13: E-value anlayses: well done!
Response 13: Thank you!

Comment 14: Please correct typos (e.g., „cognative ability")
Response 14: Thank you for pointing out this error. We have now corrected it.

Comment 15: Strengths and limitations: Mainly appropriate.
Response 15: Thank you!

Comment 16: Discussion: Please focus on the magnitude of the associations.
Response 16: We have checked and revised the Discussion accordingly to make sure discussions have focused on the magnitude of the observed associations. For example, "Chronic loneliness is recognised as a risk factor for premature death [8], with greater health risks than transient loneliness. Our findings indicate that chronic loneliness potentially accelerate physical, mental and behavioural health risks. This echoes previous research demonstrating that social deficits worsen health via unhealthy behaviours, stress and psychological stressors, and physiological repair mechanisms [24]. However, no evidence was identified for isolation in our findings, suggesting that the potential health impacts of subjective loneliness and objective social isolation could be explained through different mechanistic pathways [8]. When examining the transient phenotype, we found that transitioning out of isolation was associated with improved cognitive function, but no evidence emerged for functional improvements after loneliness remission. This differential pattern could be partially explained through the distinct neurobiological mechanisms for loneliness and isolation [40]. Chronic loneliness may primarily impact health through sustained stress responses [41], whereas remission is associated with

reduced chronic inflammation that interferes with cognitive health [32]. Remission may also benefit cognitive health via acute attentional and motivational improvements although these could have a long-lagged effect, warranting further investigations with longer follow-up periods. In contrast, neural changes related to social isolation may be reversible [42] , and isolation remission may provide additional cognitive stimulation from new social interaction [43,44]. Overall, these patterns underscore the conceptual distinction between objective isolation and subjective loneliness, suggesting that future research should explore their distinct mechanisms when designing targeted interventions. " (Page 12)

Comment 17: Conclusion: Please tone down the wording. Moreover, please tone down the practical implications. Reverse causality cannot be ruled out.
Response 17: We have now revised the conclusion to make sure we are not overstating our findings: "Using an outcome-wide longitudinal approach, this study assessed how chronic phenotypic loneliness and isolation, as well as dynamic transitions between temporary phenotypes, are associated with physical, mental and cognitive health of older adults. We identified a potential dose-response relationship between chronic loneliness and adverse health outcomes, indicating the potential for cumulative adverse health impacts of chronic loneliness, particularly for mental and physical health. Effect sizes for the onset of loneliness are similar across these domains, yet attenuated relative to the chronic phenotype. In contrast, social isolation was primarily associated with mental health outcomes, and there was some indication that transitioning out of isolation may be linked to improvements in cognitive health. These findings supplement growing evidence that chronic loneliness may have a key public health role in later-life health across physical, mental and behavioural health domains, with variation across chronic phenotypes and dynamic transitions. Future research should further elucidate the temporal dynamics underlying these observed patterns and explore whether interventions focused on the specific nature and duration of social deficits - including chronic phenotypes and transitions between temporary phenotypes - can optimise targeted prevention and treatment strategies for supporting healthy ageing." (Page 14).

Comment 18: Methods: Please add response rates for the waves used (and attrition rate)
Response 18: We have now added response rates to the Methods section: "In this study, we used ELSA data from the most recent six-year period, including wave 6 (2012-2013; response rate = 71.8%-81.8%), wave 7 (2014–2015; response rate = 78.3%-81.4%), wave 8 (2016–2017; response rate = 82.4%) and wave 9 (2018–2019; response rate = 79.5%)." (Page 14)

Comment 19: Methods: Why just a binary indicator of chronic conditions? (most older individuals have at least two chronic conditions)? Why only these few chronic diseases?
Why just a single-item to quantify pain?
Response 19: In this secondary data analysis, we used the most rigorous measures available in ELSA and those most commonly adopted in previous research. Because we were limited to existing ELSA data, we were not able to incorporate measures that are not collected in the study. The binary indicator of chronic conditions and the set of chronic diseases included were pre-defined within ELSA and have been widely used in earlier research (e.g. https://doi.org/10.1038/s41591-023-02506-1).

We agree that most older adults have multiple chronic conditions and that a more granular measure (e.g. a comorbidity count or index) can be informative. In the present study, we opted for a binary indicator for several reasons. First, there were data and measurement constraints: chronic conditions were captured in a way that did not permit construction of a reliable, standardised comorbidity index. Several diagnoses had substantial missingness or inconsistent coding across waves, which would have introduced non-random measurement error into a count or weighted index. Second, given our sample size and the number of covariates already included, a simple binary indicator (any chronic condition vs none) allowed us to adjust for the overall presence of chronic illness while preserving statistical power and avoiding perfect prediction. Our primary aim was to identify the presence of any chronic conditions rather than to explain variation in multimorbidity itself.

ELSA includes a validated single item on chronic pain: at each interview, participants were asked whether they were "often troubled with pain" and, if so, how severe the pain was (mild, moderate, severe). ELSA

does not routinely assess pain duration, and no multi-item pain scales were available. Single-item pain measures are widely used in large epidemiological surveys and have demonstrated acceptable validity for capturing overall pain burden at the population level (doi.10.1016/j.eclinm.2025.103209). We have added the reference to the pain measure [ref 50].

Comment 20: Please add psychometric properties for the cognitive function tool.
Response 20: Thank you for this helpful suggestion. We have now added information on the psychometric properties of the cognitive function measures used in our study. In the Methods section, we have now clarified that the cognitive function tool used in ELSA has demonstrated good reliability and validity: "These tests have been widely used in longitudinal ageing studies, and their reliability and construct validity have been well documented [59–62]." (Page 15). We have cited the relevant validation studies to support these statements and to provide readers with additional detail on the development and psychometric evaluation of the ELSA cognitive measures (Refs: 58, 59-62).

Comment 21: Depression: Please change to probable depression. This is just a screening tool.
Response 21: We have now revised depression to probable depression throughout discussion, and we also refer the reviewer to Response 10.

Comment 22: Life satisfaction: Why just a single-item tool? Why dichotomize it?
Response 22: Single-item measures have been shown to have acceptable reliability and good construct validity (doi: 10.1007/s11136-014-0726-4), and our choice was constrained by what is available in the ELSA dataset (ref 66-67). We dichotomised the measure to enhance interpretability and policy relevance by distinguishing individuals with low versus high life satisfaction, enabling clearer communication of findings. Given the skewed distribution of responses on the continuous 7-point scale in our sample, it's not feasible to include a sensitivity analysis.

Comment 23: Health behaviors: Why outcomes? Could these not rather be potential mediators?
Response 23: The primary aim of this study was to examine the mental, physiological, behavioural, and cognitive health consequences of chronic loneliness and isolation phenotypes, as well as transitions between loneliness/social isolation states. We adopted an outcome-wide approach, in which health behaviours were treated as one set of outcomes alongside other health domains. We agree that health behaviours could also act as potential mediators on the pathway between loneliness/isolation and other health outcomes, as well as mediating the pathway between loneliness and mortality. However, mediation analyses were beyond the scope of the current outcome-wide design.

Comment 24: Covariates: Why these specific covariates? This remains unclear. Why not adjusting for, e.g., personality?
Response 24: We chose to adjust for a core set of sociodemographic confounders commonly included in epidemiological studies of loneliness and social isolation (e.g. age, sex, ethnicity, educational level, marital status, total net non-pension wealth, and employment status) and those available in ELSA data. The same set of covariates was used for all outcomes to maintain consistency across models. As we used a wide-outcome approach with multiple different outcomes, we limited the number of covariates to avoid over- adjustment. To assess the potential impact of unmeasured confounding (including personality), we conducted sensitivity analyses using E-values to evaluate the robustness of our findings.

Comment 25: Statistical analysis: „All models adjusted for… and also account for reach outcome from pre-baseline".The use of lagged dependent variables (LDVs) as covariates in regression models can lead to bias (Nickell Bias). Please justify this choice.
Response 25: Our study uses an outcome-wide longitudinal design, in which we accounted for each outcome from pre-baseline (wave 6). This approach was chosen to help reduce the risk of adjusting for potential mediators [73] and to address reverse causality.
This strategy follows the methods proposed for outcome-wide longitudinal analyses [Ref 73. VanderWeele TJ, Mathur MB, Chen Y. Outcome-Wide Longitudinal Designs for Causal Inference: A New Template for Empirical Studies. Statistical Science. Published online 2018.

https://api.semanticscholar.org/CorpusID:88517950], as well as similar articles that have applied this approach: (https://doi.org/10.1038/s41467-024-45877-w; https://doi.org/10.1371/journal.pmed.1004365). We acknowledge the general concern about bias when using lagged dependent variables in certain panel data settings (e.g. panel data with fixed-effects estimators leading to Nickell bias). In our case, we use repeated-measures logistic and linear regression with adjustment for the pre-baseline outcome, rather than dynamic fixed-effects panel models, which limits the relevance of classic Nickell bias.

Comment 26: 25.8% (missingness, gait speed). This can be a serious issue. Please describe the MI approach in much more detail. Why do so many people not disclose their education or alcohol consumption? Why isn't the syntax provided by default?
Response 26: We agree that the level of missingness in gait speed is substantial. To address this, we applied a standard multiple imputation by chained equations approach, and all analyses were based on 50 weighted, imputed datasets. We have now added more details and references to the Methods section: "The percentage of missingness in each variable ranged from 0% to 25.8% (**Supplementary Table 1**). All study outcomes, exposures and longitudinal survey weights were included in multiple imputation models, using ordinary least squares (OLS) linear regression, truncated regression, ordered logistic, and logistic regression appropriate to variable types. Datasets were imputed using a multiple imputation by chained equations procedure, and 50 datasets were generated, with estimates combined using Rubin's rule [74]." (Page 17).

This study is a secondary analysis of ELSA data. Non-disclosure of variables such as education and alcohol consumption is relatively common in large cohort studies and may partially reflect social desirability bias (particularly for alcohol use) or uncertainty about exact response categories. The supporting documentation for ELSA (e.g., codebooks, user guides, and survey reports) has been provided by the ELSA team and is available elsewhere (https://www.elsadiabetes.nhs.uk/). We have now acknowledged in code availability: "No custom code was developed. All analyses were conducted using open-source Stata syntax. Code is available from the corresponding author upon request." (Page 18)

Comment 27: Supplementary Tables are helpful. Thank you. Please also report the covariates there.
Response 27: Thank you for this suggestion. We report the proportion of missingness in the covariates in Table 1. In the supplementary tables, we present the associations between the chronicity of loneliness and isolation and subsequent physical and mental health outcomes in later life, including effect sizes for all outcomes. Because the covariates were included as confounders rather than primary variables of interest, we did not report their individual estimates in the supplementary tables, which is consistent with common practice in the literature to avoid the Table 2 fallacy.

Reviewer #2:

Comment 1: Thank you for the opportunity to review the paper entitled 'Chronic loneliness and isolation phenotypes and physical and mental health in older adults'. The study aims to assess the association between chronic loneliness and chronic social isolation and 17 health outcomes. It adds to the growing body of research which focuses on chronic loneliness, in particular. The exposures, chronic loneliness and chronic social isolation, were defined as being lonely or isolated at two consecutive data time points. While there is not an agreed upon measure of chronic or transient loneliness or isolation, prior research generally refers to chronic loneliness or isolation as being persistent, lasting for several years or more or at consecutive time points.

Chronic loneliness was associated with increased odds of poorer health, mental wellbeing, functional capacity and health behaviours. Chronic isolation was associated with lower quality of life, and transient or remitting social isolation was associated with better mental wellbeing and cognitive function. Supplementary analysis indicates that the main results hold when both loneliness and isolation are included in the same model.
Response 1: We are grateful for the positive feedback from the reviewer.

Comment 2: I just have a few additional comments:
Most of the focus of the introduction is chronic loneliness. More could be developed about social isolation and the potential implications of chronic social isolation.
I suggest the authors consider some of the recent papers on chronic loneliness and chronic social isolation (see for example, the review by Hajek et al. (2025). Chronic loneliness and chronic social isolation among older adults. A systematic review, meta-analysis and meta-regression, Aging & Mental Health, 29:2, 185-200, DOI: 10.1080/13607863.2024.2385448; Lim, M.H., Manera, K.E., Owen, K.B. et al. (2023) The prevalence of chronic and episodic loneliness and social isolation from a longitudinal survey. Scientific Reports, 13, 12453. https://doi.org/10.1038/s41598-023-39289-x)
Response 2: Thank you for the reviewer's comment and references. We have now incorporated the review by Hajek et al. (2025) and the paper by Lim, M.H.(2023), and new references on social isolation to strengthen our papers. We have now revised and expanded the background to provide a more comprehensive discussion of social isolation throughout the introduction (Pages 2-3).

Comment 3: The findings for social isolation and health behaviours are not commented on.
Response 3: Thank you for this helpful comment. We have now clarified the findings regarding social isolation and health behaviours in the Results section by adding the following sentence: "In contrast, isolation phenotypes were not associated with health behaviour domains." (Page 8)

We also further contextualise these findings in the discussion: "Both chronic and incident isolation phenotypes were associated with lower QoL, though not with other health domains, including health behaviours. In contrast, transitioning out of isolation was associated with reduced odds of probable depression, greater odds of reporting happiness, and with better cognitive health." (Page 12)

Reviewer #3:

Comment: I am afraid that i found the paper somewhat hard to understand. It would help if the Methods section was in the correct place - it appears to be placed after the discussion. However, I think what is missing is a clear definition of the exposures - chronic loneliness and chronic social isolation - transient loneliness etc. You need to say how these were determined and analysed. It would then also help if you provided very clear research questions and/or hypotheses.
Response: Thank you for your comments. Regarding the structure of the manuscript, the Methods section is placed after the Discussion in accordance with *Nature Communications* journal formatting guidelines. We have now added hypotheses following the research aims in the introduction: "Thus, this study used an outcome-wide approach to assess the physical, mental, behavioural, and cognitive health consequences of chronic loneliness and isolation phenotypes and the transitions between loneliness/social isolation states (i.e. onset and remission) over two years. We hypothesised that chronic and onset of loneliness/isolation would be associated with worse health outcomes, whereas remission would be linked to improvements." (Page 3)

We have now added new statements to clarify datasets and data analyses at the beginning of Results: "We analysed panel data from the English Longitudinal Study of Ageing (ELSA) from 2012 to 2018, examining how chronic and transient social deficits were associated with physical, mental, cognitive, and behavioural health outcomes using regression models. The study included 4,090 participants with a mean age of 66.7 years (SE=0.13, age range= 54-89 years) (Table 1)." (Page 3).

We have now clarified how we defined and measured the exposures in the Results: "We defined chronic phenotypes as loneliness/isolation reported consistently across two consecutive waves. Onset was defined as reporting loneliness or isolation at the first wave only, whereas remission was defined as solely reporting loneliness or isolation at the second wave." (Page 4) We have also improved our definitions of chronic vs transient social deficits in the introduction: "Chronic loneliness and isolation phenotypes present as a persistent, lasting emotional state of feeling lonely or disconnected over an extended period of time [5,8,18–20]. In contrast, transient loneliness or isolation represents a subacute phenotype, with different health consequences compared to the persistent trait [21]. Although there is no consensus threshold for the chronic

phenotype, studies commonly define it as loneliness or isolation reported consistently across repeated measurements over years or at consecutive time points [5,8,20] ." (Page 2)

In the Methods (Exposures) section, we also have clarified how these constructs were operationalised and analysed:
"Loneliness was measured using the 3-item UCLA scale [69], with a sum score ranging from 3 to 9 and a cut-off score of ≥6 used to indicate loneliness [70,71]. Social isolation was measured using the five-item Steptoe Social Isolation Index by combining isolation in marital status (unmarried or living alone), less than monthly contacts with children, relatives, or friends, and no social engagement in any groups, clubs or other social organisations, with an overall score ranging from 0 to 5. We applied a cut-off at ≥3 to indicate social isolation [3,71,72]. We used these measures across waves 7 and 8 to generate two four-category summaries of sustained social deficits over two years: none (not lonely at wave 7 and 8), transition out of loneliness (remission, lonely at wave 7 and not at wave 8), onset of loneliness (not lonely at wave 7 and lonely at wave 8), and chronic loneliness (lonely at wave 7 and 8). We repeated this procedure for social isolation." (Page 16)

Reviewer #4:

Comment 1: This manuscript provides a comprehensive analysis using ELSA data to study the associations between chronic loneliness and isolation and physical and mental health in older adults. Overall, the manuscript effectively reflected on prior literature and addresses an important topic substantively. Below, I provide a mix of major and minor critiques for your consideration.
Response 1: We are grateful for the positive feedback from the reviewer.

Comment 2: Major Comments:1. In the Background (p.2), the authors suggest that "older people are particularly vulnerable to loneliness". However, the youngest age groups are more likely to report loneliness than other age groups (e.g., Office for National Statistics, 2025). Although, older people are more likely to have experiences that lead to loneliness, authors need to make more accurate claims. Authors also suggested that "health effects of loneliness are more prominent among older adults" (p.2). Can the authors provide a reference to support this claim?
Response 2: Thank you for recognising this. We have now revised the statement in the Background to "Older people are vulnerable to social deficits (loneliness or social isolation)", which has been widely supported in previous literature [3,4,5]. This phrasing more cautiously reflects the evidence and does not imply that older adults are the most lonely age group.

We agree that younger age groups often report higher levels of loneliness than older adults (e.g. Office for National Statistics, 2025). Our intention in the opening paragraph was not to compare age groups directly, but to highlight loneliness as a key issue in later life. To better reflect the life-course pattern, we have now explicitly acknowledged that: "Loneliness follows a U-shaped distribution across the lifespan, being more prevalent among younger adults and steadily prominent in older adults aged 60 years and above, with its adverse health effects particularly pronounced in older adults [6–8]. Similarly, social isolation tends to increase as individuals age, with the oldest adults facing a higher risk of living with social isolation compared to those under 70 years [8,9].". We have also added references to support these statements (References 6-9).

Comment 3: The mechanisms behind the associations between loneliness and physical health are discussed (p.2). As the manuscript also focuses on mental health, can the authors discuss the ways how chronic loneliness may affect mental health (i.e. depression and cognitive function changes)?
Response 3: Thank you for your comments. In the Introduction, the mechanisms we outlined could link loneliness to both mental and physical health. We have therefore clarified that "adverse health impacts" include both physical and mental health. This also covers mechanisms of social deficits and mental health (i.e. depression and cognitive function changes), and we highlight epidemiological evidence linking loneliness to anxiety, depression, and dementia in paragraph 1.

Comment 4: It would be helpful to provide a figure that makes it clear what variables were measured at each wave.
Response 4: Thank you for the suggestion. As recommended, we have added Supplementary Figure 1 to clearly show when each variable included in our study was measured. We have summarised and referenced this figure in the main text: "To form our final analytical sample, we used waves 7 (2014/2015) and 8 (2016/2017) as our exposure window to measure changes in social deficits over two years. Health outcomes were measured at wave 9 (2018/2019) for longitudinal analyses. Confounders were measured in the wave preceding the start of the exposure window (wave 6; 2012/2013) (**Supplementary Figure 1**)." (Page 14).

Supplementary Figure 1. Measurement of variables across waves

	Pre-wave (wave 6)	Pre-wave (wave 7)	Baseline (wave 8)	Follow-up (wave 9)
**Sociodemographic characteristics**				
Age	X			
Sex	X			
Ethnicity	X			
Education	X			
Employment	X			
Marital status	X			
Total (non-pension) wealth	X			
**Exposures**				
Loneliness		X	X	
Isolation		X	X	
**General health**				
Self-rated health	X		X	X
Number of chronic illnesses	X		X	X
Frequent pain	X		X	X
**Functional capacity**				
ADLs disability	X		X	X
IADLs disability	X		X	X
Mobility disability	X		X	X
Gait speed	X		X	X
**Cognitive function**				
Cognitive functional score		X	X	X
**Mental wellbeing**				
Depressive symptoms	X		X	X
Life satisfaction	X		X	X
Happiness	X		X	X
Quality of life	X		X	X
**Health behaviour**				
Sleep problems	X		X	X
Sedentary behaviour	X		X	X
Smoking	X		X	X
Alcohol consumption	X		X	X
Leisure engagement	X		X	X

Comment 5: The cut-off of ≥3 for CES-D score was used in the study to indicate depression. Many studies have also used cut-off ≥4 to indicate potential depression (e.g.,Beach et al., 2003, PLOS Medicine). Have authors conducted the analysis with cut-off 4 to compare if there were any differences in the results?
Response 5: The eight-item Center for Epidemiologic Studies Depression Scale (CES-D) is a well-established, validated measure that is widely used in survey and population-based studies of late-life depression. The cut-off of ≥3 for the eight-item CES-D is the most commonly used threshold, particularly in general population and older adult samples (including ELSA, and the Health and Retirement Study, etc.), as it provides a good balance of sensitivity and specificity, as described in the following studies:

- Karim, J., Weisz, R., Bibi, Z. et al. Validation of the Eight-Item Center for Epidemiologic Studies Depression Scale (CES-D) Among Older Adults. Curr Psychol 34, 681–692 (2015). https://doi.org/10.1007/s12144-014-9281-y
- Schlechter P, Ford TJ, Neufeld SAS. The Eight-Item Center for Epidemiological Studies Depression Scale in the English Longitudinal Study of Aging: Longitudinal and Gender Invariance, Sum Score Models, and External Associations. Assessment. 2023 Oct;30(7):2146-2161. doi: 10.1177/10731911221138930.
- Gao Q, Steptoe A, Fancourt D. Chronic loneliness and isolation phenotypes, incident functional impairment and mortality in England 397 between 2004 and 2018. Nature Mental Health. Published online 2025. 398 doi:10.1038/s44220-025-00436-0

However, we tested a cut-off of ≥4 and observed no significant differences in the results using complete cases or following imputation; our conclusions remained unchanged. Here are the results using a cut-off of ≥4 in weighted and imputed datasets, compared to the original cut-off of ≥3:

Longitudinal Associations Between Chronicity of Loneliness and Isolation and Subsequent Depression

**Outcomes**	Transition out of loneliness (ORs)	Onset of loneliness (ORs)	Chronic loneliness (ORs)
Depression (CESD ≥3)	**1.78 [1.24-2.54]**	**1.98 [1.39-2.84]**	**4.39 [3.36-5.74]**
Depression (CESD ≥4)	**1.72 [1.13-2.63]**	**2.18 [1.44-3.31]**	**5.63 [4.18-7.59]**
	Transition out of isolation (ORs)	Onset of isolation (ORs)	Chronic isolation (ORs)
Depression (CESD ≥3)	**0.69 [0.54-0.88]**	1.20 [0.80-1.80]	0.99 [0.77-1.28]
Depression (CESD ≥4)	**0.60 [0.45-0.82]**	1.41 [0.88-2.27]	1.11 [0.82-1.49]

Note. Reference category no loneliness/isolation. Bold text indicates $p<0.05$. Results were weighted and based on 50 imputed datasets. OR: odds ratio. All tests were two-sided.

Comment 6: Minor comments: When discussion the limitations, the authors could also mention healthy survival bias, which is relevant for the study.
Response 6: We have now added survival bias into the limitations, as a type of selection bias:
"We limited the sample to those who attended continuous waves of surveys for analytical purposes, which may introduce selection bias (e.g. healthy survival bias)." (Page 13)

Comment 7: Can the authors provide the references for the use of ADLs, IADLs, functional difficulties, and sedentary behaviour measures (p.14)?
Response 7: We have now added references for the ADL, IADL, functional difficulties, and sedentary behaviour measures on p.14. These are cited as References 51-54 and 68 in the revised manuscript.

Comment 8: In the sensitivity analysis section (p. 7), can the authors provide a reference to support the claim that the results were moderately robust to unmeasured confounding, according to the calculated E-values?
Response 8: We have now added references to support the interpretation of E-values (References 75-76).

Comment 9: In the Supplementary Figure 1, the number of participants not included in all waves seems to be missing.
Response 9: In Supplementary Figure 1, we included the category "sample excluded – not included in all waves" at the initial step but did not provide a single number, as Waves 6-9 have different sample sizes. We have now added the aggregate number of participants not included in all waves to the Supplementary Figure.

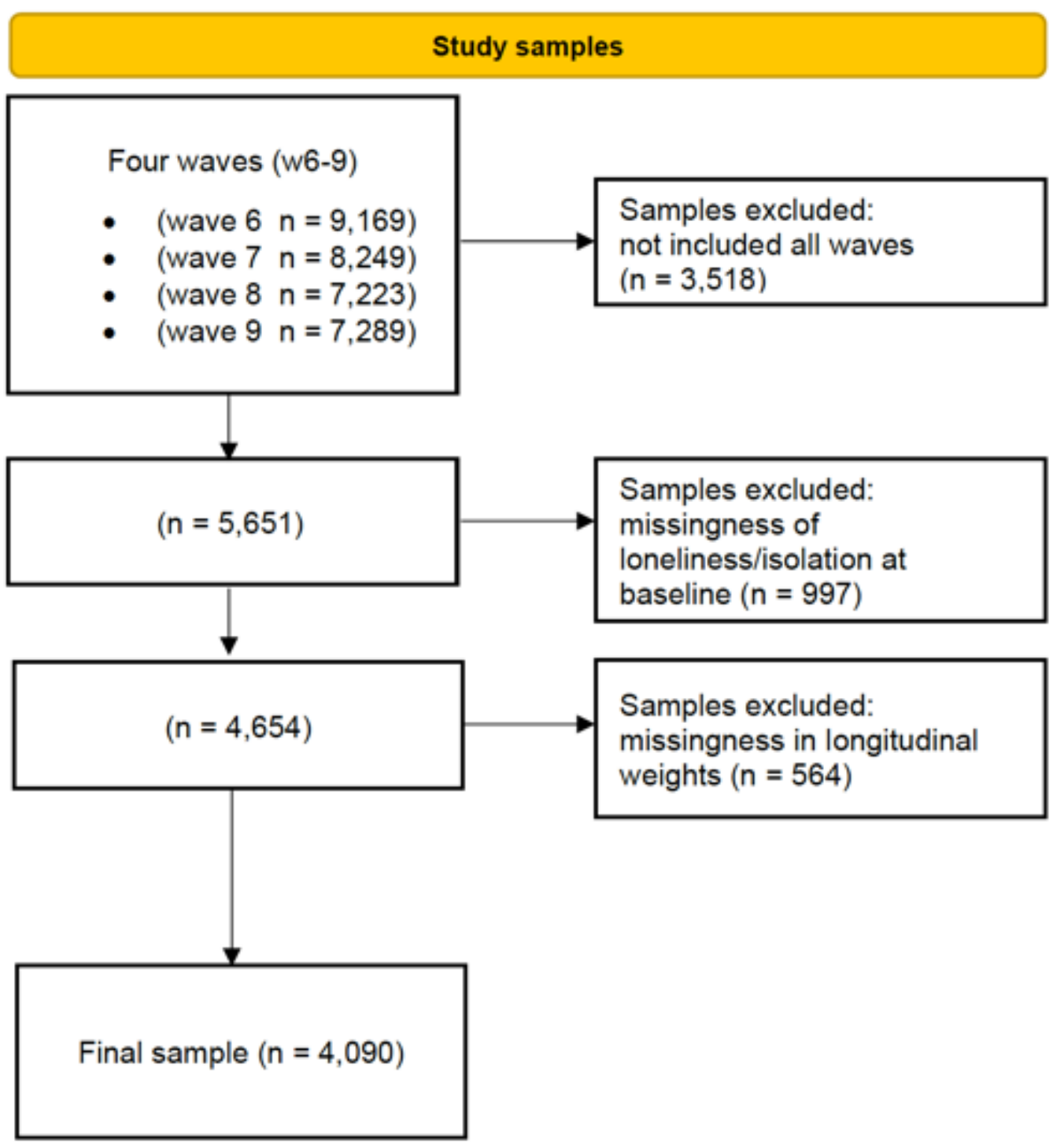

Dear Editor,

Thank you for the additional opportunity to revise our manuscript.

We are grateful to the editor and reviewers for their further encouraging and insightful comments, all of which we have carefully attended to and responded to below. We therefore hope this paper is now suitable for acceptance.

The authors

REVIEWER COMMENTS

Reviewer #1

Comment 1: For the most part, the issues were addressed effectively.
Response 1: Thank you, we are glad that we were able to address your comments, which definitely improved our manuscript.

Comment 2: A few issues: Abstract: The last sentence is still phrased in a very causal manner. Please revise it further. Endogeneity cannot be ruled out. The conclusions are also too strong.
Response 2: We have now revised it as: "Findings suggest a potential dose-response relationship between chronicity of loneliness and adverse health outcomes, highlighting chronic social deficits as an important correlate of healthy ageing."

Comment 3: Conclusion (text): Please tone down (see my comment above).
Response 3: We have carefully reviewed throughout to avoid causal language and have now revised it as "Using an outcome-wide longitudinal approach, this study assessed how chronic phenotypic loneliness and isolation, as well as dynamic transitions between temporary phenotypes, are associated with physical, mental and cognitive health of older adults. We identified a potential dose-response relationship between chronic loneliness and adverse health outcomes, particularly for mental and physical health. Effect sizes for the onset of loneliness are similar across these domains, yet attenuated relative to the chronic phenotype. In contrast, social isolation was primarily associated with mental health outcomes, and there was some indication that transitioning out of isolation may be linked to improvements in cognitive health. These findings supplement growing evidence that chronic loneliness is linked to later-life health across physical, mental and behavioural health domains, with variation across chronic phenotypes and dynamic transitions. Future research should further elucidate the temporal dynamics underlying these observed patterns and explore whether chronic social deficits might represent a target for intervention to improve healthy ageing."

Comment 4: Lagged dependent variable: Please consider omitted variable bias. The LDV is very likely correlated with unobserved heterogeneity (e.g., personality, genetic factors). The model then incorrectly attributes an influence to the lagged value that actually stems from these stable factors. Please extend the limitations section accordingly (or modify the model)
Response 4: Thank you! We agree that, although adjusting for pre-baseline values of the outcome helps account for prior health status and reduces the possibility of reverse causation, lagged dependent variable models do not fully eliminate the risk of omitted variable bias.

As the primary aim of this study was to estimate prospective associations between chronic loneliness/social isolation phenotypes and a broad range of later-life health outcomes, rather than to establish causality, we have retained the original modelling strategy to fit our outcome-wide approach. However, we have expanded the Limitations section to acknowledge this issue: "We adjusted for sociodemographic confounders and accounted for pre-baseline outcomes to minimise the risk of adjusting for potential mediators and address reverse causality. Nevertheless, the possibility of omitted variable bias cannot be ruled out. Future studies using causal inference approaches are warranted to further investigate these findings." (Page 13)

Comment 5: Why isn't the Stata code (I don't mean the DTA file) made available (but only upon request)? Unfortunately, this question isn't clearly answered. In the spirit of open science, this would be highly desirable.
Response 5: Apologies that we missed this before; all code for analyses in this study is publicly available online: https://github.com/ytmdata/project. (Page 18)

Reviewer #2

Comment: The authors have addressed my previous comments and the paper has improved.
Response: Thank you!

Reviewer #3

Comment 1: I have several concerns about this paper:
You ought to demonstrate how this study clearly differs from other studies of loneliness and health using the ELSA data. I have a strong sense of deja vu; I feel I have seen many of these findings before - thus, you need to state what is different or innovative.
Response 1: While previous research using ELSA and other cohorts has examined loneliness/ isolation and health outcomes, most studies have focused on single outcomes and have not considered the duration and persistence of social deficits. In theory, compared to transient phenotypes or no loneliness/isolation, chronic phenotypes may accumulate more adverse health impacts through interconnected biological, behavioural, and psychological pathways. Chronic loneliness/isolation may have distinct implications from transient or occasional experiences. According to a very recent systematic review published in 2025 (reference 5) on chronic loneliness and social isolation, there is relatively little research examining chronic loneliness and isolation.

To our knowledge, only one previous study using ELSA data (reference 8) investigated the role of chronic loneliness and isolation in predicting mortality as well as the onset of difficulties with activities of daily living (ADL) as a competing outcome in modelling. Our study builds on this existing evidence by exploring the various physical, mental, and behavioural pathways that might underlie the association between chronic loneliness and mortality. We use an outcome-wide approach to look at a diverse range of physical, mental, and behavioural factors, taking the first step in identifying which are associated with chronic loneliness. This could lead to future research exploring whether specific factors are mediators of the association between chronic loneliness and mortality. We are testing a range of factors that could represent potential mechanisms. The value comes both from 1) identifying the outcomes of chronic phenotypes so that we better understand how chronic loneliness/isolation might be influencing health, and 2) identifying potentially modifiable mediators of the relationship between chronic loneliness/isolation and mortality that could be further tested in future research and ultimately intervened on. We therefore believe that this study makes a timely and novel contribution to the literature on chronic social deficits and healthy ageing.

We have added more information on the novelty of our study in paragraph 3 of the introduction (Page 3):
"Given the evidence, there are increasing concerns about the impacts of social deficits on public health [37]. However, previous research often measures social deficits at a single timepoint, with limited attention to the dynamic transitions into and out of loneliness/isolation or long-term exposure to social deficits. Transient loneliness/isolation may have different health impacts compared to chronic phenotypes [38]. These phenotypes are theoretically distinct [39]. A recent systematic review highlighted the need for further longitudinal investigations, particularly on chronic loneliness and isolation[5]. Notably, it remains unclear whether experiencing social deficits chronically has cumulative health risks across diverse health domains. Although chronic loneliness and social isolation have been identified as independent risk factors for premature mortality in older age [8,15,23], the physical, mental and behavioural pathways underlying the associations between chronic social deficits and premature mortality have yet to be fully explored [5]. Further research

should build on this evidence by testing a range of factors that could represent potential mechanisms. This could help explore the health outcomes of chronic phenotypes and identify potential modifiable mediators of their association with mortality that could be further examined and ultimately intervened on."

Comment 2: There is no methods section to state how you arrived at your findings.
Response 2: The Methods section is presented after the Discussion section (pages 14-18), as required by Nature journals.

Comment 3: The research questions are not stated. There are no hypotheses to guide this work.
Response 3: We have now clarified both the study questions and hypotheses in the revised manuscript.

Specifically, we added the following hypothesis: "We hypothesised that chronic and incident loneliness/isolation would be associated with worse health outcomes, whereas remission would be linked to improvements. Chronic phenotypes would be worse than onset, showing a dose-response relationship." (Page 3)

We have also explicitly stated the research question before the hypothesis in the Introduction: "Thus, this study used an outcome-wide approach to assess the physical, mental, behavioural, and cognitive health consequences of chronic loneliness and isolation phenotypes and the transitions between loneliness/social isolation states (i.e. onset and remission) over two years. Our research questions were (i) are chronic loneliness and isolation phenotypes and the transitions between loneliness/social isolation states related to poorer health outcomes in older age? (ii) what are the relationships for physical, mental, behavioural, and cognitive health outcomes? (iii) is there a dose-response relationship between chronicity of loneliness/isolation and health outcomes?" (Page 3).

Comment 4: Might be useful to offer a power calculation.
Response 4: We appreciate the reviewer's suggestion regarding a power calculation. However, because this study is a secondary analysis of an existing cohort dataset (ELSA), the sample size was determined by the number of eligible participants available in the dataset rather than by prospective recruitment. Formal a priori power calculations are generally most relevant to studies in which the sample size can be planned and adjusted before data collection (references 1-2). ELSA is a large nationally representative dataset. In our analysis, we included all participants meeting the predefined eligibility criteria to maximise statistical precision and minimise selection bias. We have therefore not added a power calculation. Nonetheless, the precision of the reported effect estimates (as reflected in the confidence intervals) provides information regarding the statistical uncertainty of our findings (reference 1).

References

1 : Hernán, M. A. (2022). Causal analyses of existing databases: no power calculations required. *Journal of clinical epidemiology*, 144, 203-205.
2 : Dziak, J. J., Dierker, L. C., & Abar, B. (2020). The interpretation of statistical power after the data have been gathered. *Current Psychology*, 39(3), 870-877.

Comment 5: minor but important point - never use the term "non-white" - first this is not an ethnic category and second, it always feels pejorative.
Response 5: We agree with the reviewer. We have now revised the terminology throughout the manuscript and replaced "non-White" with "other ethnic groups" where appropriate.

Reviewer #4

Comment: Thank you for addressing the comments. I am satisfied with the responses and revisions made to the manuscript.
Response: Thank you!

Dear Editor,

Thank you for the additional opportunity to revise our manuscript.

We are grateful to the Editor and Reviewers for their insightful comments, all of which we have carefully attended to and responded to below. We hope that the revisions have addressed all remaining concerns, and the manuscript is now suitable for publication.

Yours sincerely,
The authors

REVIEWERS' COMMENTS

Reviewer #1

Comment: Thank you for further improving the paper. However, one point definitely needs to be worded more carefully. More precisely, please avoid the term "dose-response relationship" (which sounds very causal) in the abstract and the conclusion (main text). For example, you could rephrase it to: "is associated with"

Response: Thank you for this helpful suggestion. We have now revised the wording in both the Abstract and the Conclusion to use more appropriate associative language:
Abstract (Page 1) “Findings suggest an association between chronicity of loneliness and adverse health outcomes, highlighting chronic social deficits as an important correlate of healthy ageing.”
Conclusion (Page 14) “We found that chronic loneliness was associated with adverse health outcomes, particularly for mental and physical health.”

Reviewer #3

Comment: I am satisfied that the authors have addressed the main comments.

Response: Thank you!
